# Novel Animal Models for Multiple Sclerosis: R-Ras GTPases in Myelin Pathophysiology

**DOI:** 10.3390/biom15091309

**Published:** 2025-09-11

**Authors:** Gema M. Esteban-Ortega, Gonzalo Garcia-Martin, Beatriz Cubelos

**Affiliations:** 1 Centro de Biología Molecular Severo Ochoa (CBM), Universidad Autónoma de Madrid, 28049 Madrid, Spain; gema.esteban@uam.es (G.M.E.-O.); gonzalo.garciam@uam.es (G.G.-M.); 2 Instituto Universitario de Biología Molecular, Universidad Autónoma de Madrid, 28049 Madrid, Spain

**Keywords:** neurological disorders, neuroimmunology, neuroinflammation, myelination, oligodendrocyte, multiple sclerosis, R-Ras related, oligodendroglial maturation, preclinical trials, molecular targets, therapeutic strategies

## Abstract

Demyelinating diseases, such as multiple sclerosis, involve oligodendrocyte death, myelin loss, and neuronal death. These processes have been extensively studied, and a causal relationship has been demonstrated between them: destruction of oligodendrocytes results in myelin deficiency, which subsequently leads to neurodegeneration and the consequent loss of sensory, motor, and cognitive functions. Currently, myelinopathies lack fully effective treatments. Available drugs primarily focus on controlling the immune response without directly promoting myelin regeneration or restoring neuronal functionality. Alongside these treatments, pharmaceutical research has increasingly focused on developing therapies that stimulate oligodendroglial lineage differentiation and myelin sheath regeneration. Despite these advances, the lack of suitable preclinical models has been a significant obstacle in evaluating new therapeutic compounds. In this review, we present the main animal models used in the preclinical phase for the study of myelin-related diseases and their role in the development of new therapies. In addition, we highlight the usefulness of R-Ras animal models for assessing the efficacy of compounds that promote oligodendrocyte differentiation.

## 1. Introduction

Myelin sheaths are lipid-rich structures that coat the axons of neurons. In addition to providing insulation, myelin sheaths are essential for maintaining axonal metabolism and the proper transmission of nerve impulses in the central nervous system (CNS) [1,2,3,4,5,6]. In this way, myelin contributes to the synchronization of electrical impulses across different regions of the nervous system [7,8,9].

Oligodendrocytes are the glial cells responsible for myelination in the CNS. During development, oligodendrocytes arise from oligodendrocyte progenitor cells (OPCs). This process, known as oligodendrogenesis, takes place in specific restricted regions along the dorsoventral axis, from where the entire CNS is eventually colonized [10,11,12,13,14,15,16,17,18,19]. As they mature, oligodendrocyte progenitor cells undergo substantial changes: from a bipolar cell with high migratory and proliferative potential to an immotile but highly branched oligodendrocyte that can generate multiple different myelin sheaths [20,21,22]. The differentiation of oligodendrocyte progenitor cells is regulated by a combination of inhibitory and inducing factors, as well as extrinsic and intrinsic signals. These signals include growth factors, protein kinases, and extracellular matrix proteins, all of which influence gene expression to modulate oligodendrocyte maturation [23,24,25].

When the process of an OPC comes into contact with the axonal membrane it will eventually cover, it stabilizes and forms a specialized domain for communication between the axon and the oligodendroglial cell. Subsequently, the myelin sheath expands radially and longitudinally, adding new membrane layers at the growth tip of the oligodendroglial process, known as the inner tongue [26,27,28]. As this progresses, the innermost layers of the sheath compact and adhere to the axon, which ultimately becomes fully enveloped.

**Figure 1:**
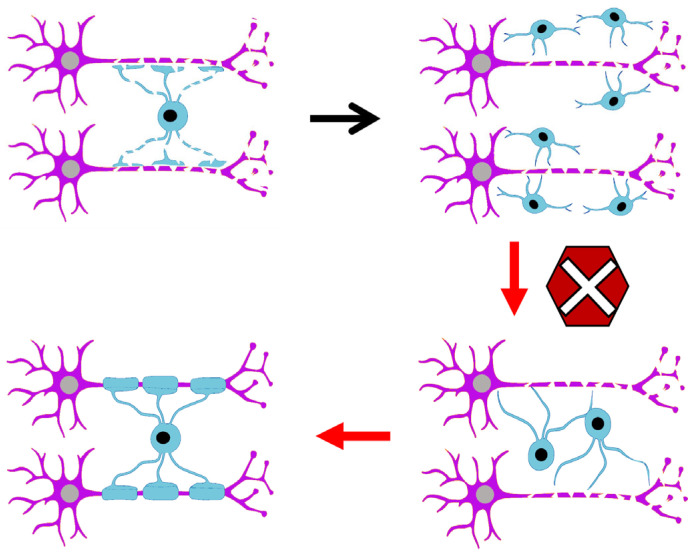
The blockage in oligodendrocyte maturation prevents effective myelination.

However, not all oligodendrocytes generated perinatally are destined for immediate myelination. Indeed, some oligodendrocytes remain undifferentiated as endogenous OPCs in the adult brain [29,30]. In the adult CNS of primates, approximately 5-10% of the total cell population consists of endogenous OPCs [31]. Their role will be focused on replacing dying oligodendrocytes to ensure an active remyelination and the maintenance of oligodendrocyte functions [32,33,34]. This is because oligodendrocytes modulate neuronal circuits throughout life, as myelin is produced continuously, and their involvement in plasticity processes supports the maintenance and acquisition of new skills [33,35,36,37,38]. Experimental evidence shows that patients with myelin-related diseases exhibit a blockage in the differentiation of oligodendrocyte progenitor cells into mature oligodendrocytes [37,39,40], suggesting that the molecular signals regulating myelination are disrupted and prevent proper myelin repair (Figure 1). Recent works highlight that the majority of differentiating OPCs do not go onto integrate successfully following differentiation [41,42]. Likewise, in multiple sclerosis lesions, the presence of premyelinating oligodendrocytes is suggestive that the initial steps of differentiation may not be impaired but instead the ability to find suitable axons to remyelinate or survival may be impacted [43,44].

Myelin disturbances are the cause of severe pathologies such as neuromyelitis optica, hypomyelinating leukodystrophies, Charcot–Marie–Tooth disease, and multiple sclerosis, among others. Generally, these diseases are characterized by the formation of demyelinating lesions, accompanied by axonal damage and an inflammatory response in the tissue. Ultimately, loss of myelin impairs nerve impulse transmission and can result in disrupted neuronal function. In advanced stages, demyelination is associated with neurodegeneration, contributing to the clinical variability and heterogeneous progression of these conditions. Depending on the impact over the nervous tissue, lesions are classified as acute plaques, chronic active plaques, or chronic inactive plaques [6,45,46,47,48,49].

Among these diseases, multiple sclerosis exhibits a higher incidence and represents the leading cause of non-traumatic neurological disability in young adults [50,51]. This disease affects more than 2.5 million people worldwide, with diagnosis typically occurring in early adulthood in most cases. Its incidence follows a bimodal pattern, with an initial peak around age 20 and a second rise around age 40. This disease not only places a significant burden on patients and their families but also on healthcare systems. Additionally, multiple sclerosis exhibits a higher prevalence in women, who account for approximately 70% of cases [52,53].

From a clinical perspective, multiple sclerosis is classified into four main forms: clinically isolated syndrome, relapsing–remitting multiple sclerosis, secondary progressive multiple sclerosis, and primary progressive multiple sclerosis [54,55].

The clinically isolated syndrome represents the first manifestation of neurological symptoms suggestive of multiple sclerosis. It typically occurs in young adults and affects the optic nerves, brainstem, or spinal cords. Following this initial episode, the disease’s progression varies: approximately one-third of patients will advance to other forms of multiple sclerosis [56,57,58]. Relapsing–remitting multiple sclerosis is the most common form of the disease and is characterized by relapses involving transient neurological deficits, followed by periods of partial or complete recovery. Over time, many patients with relapsing–remitting multiple sclerosis transition to a phase of progressive neurological decline, known as secondary progressive multiple sclerosis. On the contrary, primary progressive multiple sclerosis represents an aggressive form in which, from the onset of symptoms, there is a steady deterioration of neurological functions and a progressive accumulation of disability, without relapses or remission periods [59,60,61].

**Figure 2:**
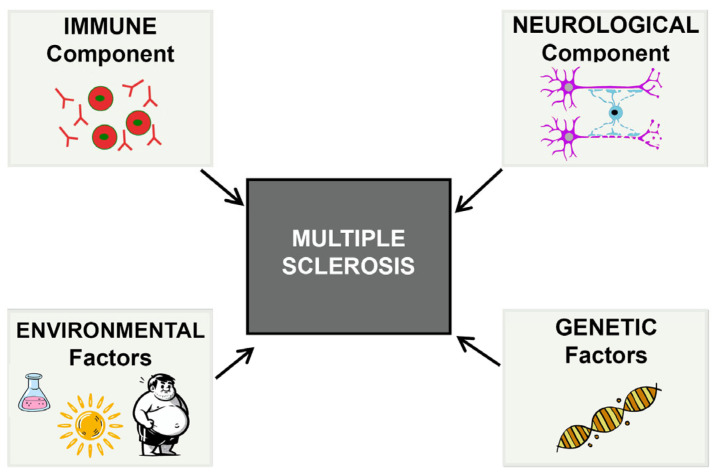
Various factors influence the onset and progression of multiple sclerosis, making this pathology a complex and multifactorial disease.

Although multiple sclerosis is a common and highly prevalent disease, its exact etiology remains incompletely understood. It is currently recognized as a chronic and complex condition involving multiple factors, with both immunological and neurological components playing prominent roles. Additionally, genetics and certain environmental factors are critical in its development [59,62,63] (Figure 2). One of the main challenges in developing drugs for multiple sclerosis is that current animal models fail to accurately replicate the complexity of human pathophysiology. These differences between the model and the human disease can limit the predictive value of preclinical results, making it more difficult to design truly effective treatments. In most cases, the models focus primarily on inflammatory processes [64] and exhibit a certain degree of spontaneous myelin regeneration, which adds another layer of difficulty in accurately assessing the therapeutic efficacy of investigational compounds.

Although significant progress has been made in the treatment of multiple sclerosis—especially with the development of disease-modifying therapies (DMTs) for relapsing–remitting multiple sclerosis, which reduce relapse rates and delay disability progression [65]—there is still no curative treatment available. These therapies act mainly by modulating or suppressing the immune response and, while effective at controlling inflammation, they do not promote remyelination or neuronal repair. This limitation has driven the development of therapies that stimulate oligodendroglial lineage differentiation and myelin regeneration, which requires preclinical models that more accurately represent human pathophysiology.

In the relapsing–remitting form of multiple sclerosis, immunomodulatory and immunosuppressive treatments such as beta interferons, glatiramer acetate, or monoclonal antibodies (natalizumab, alemtuzumab, ocrelizumab) have been shown to reduce the frequency of relapses and inflammatory activity [66]. Although disease-modifying treatments (DMTs), such as beta interferons and monoclonal antibodies, have significantly improved inflammation control and reduced relapse rates in relapsing–remitting multiple sclerosis [65,67,68], these therapies do not reverse already established neuronal damage or promote tissue repair. For this reason, current research is focused on combining therapeutic strategies that integrate immunomodulatory agents with compounds that stimulate remyelination and neuroprotection, with the goal of slowing neurodegeneration and restoring neurological function [69,70]. This multidimensional approach addresses the need to target both inflammatory mechanisms and the processes of damage and repair in the disease.

Research on multiple sclerosis has shifted toward a more neurological focus, emphasizing myelin regeneration and the restoration of oligodendrocyte function. Remyelination-oriented therapies are not a recent concept. For over a decade, strategies with this objective have been proposed, and key studies as early as 2015 demonstrated that it is possible to stimulate the differentiation of oligodendrocyte progenitors and promote myelin regeneration in experimental models [71,72,73]. Currently, this line of research is at a more advanced stage, with ongoing clinical trials, consolidating a field that has progressively evolved since then [74,75,76,77,78].

The primary challenge in developing drugs for the treatment of multiple sclerosis is the lack of animal models that accurately replicate its neurological pathophysiology. This poses a significant obstacle to creating effective treatments and finding a definitive cure. The most commonly used current models focus primarily on inflammation, as detailed in comprehensive studies such as [64,79,80,81], and allow for a certain degree of spontaneous myelin regeneration. This characteristic can hinder an accurate assessment of treatment efficacy, since natural remyelination in these models makes it more difficult to distinguish between drug effects and endogenous recovery processes.

Research in multiple sclerosis continues to advance along two complementary lines. On one hand, DMTs focus primarily on inflammation and immunosuppression to reduce disease activity and progression. On the other hand, regenerative therapies aimed at promoting remyelination and neuronal repair have coexisted with DMTs for years, seeking to address the neurological damage already established. Both approaches are necessary and complementary in the pursuit of more effective treatments that not only modulate the immune response but also restore impaired neurological function.

Currently, experimental models of multiple sclerosis can be grouped into three main categories: inflammatory models, which mimic the autoimmune response against myelin; toxic models, which induce demyelination using chemical substances; and neurological models, which replicate alterations in the development and function of oligodendrocytes. Below, the most used models in preclinical MS research will be described, highlighting their advantages and limitations.

## 2. Classical Models

### 2.1. Inflammatory Models

#### 2.1.1. Experimental Autoimmune Encephalomyelitis

The experimental autoimmune encephalomyelitis (EAE) model is one of the most widely used for studying multiple sclerosis. This model is based on the induction of an autoimmune response against myelin, which is triggered by immunization with fragments of myelin proteins or, alternatively, through the passive transfer of autoreactive T cells directed against specific myelin epitopes. More specifically, an immune response is induced in animals—mainly mice (SJL/J, C57BL/6, and BALB/c), rats (usually the Lewis rat or Dark Agouti (DA) strains), or even primates—mediated by CD4+ T cells, through immunization with myelin proteins such as myelin basic protein (MBP), proteolipid protein (PLP), or myelin oligodendrocyte glycoprotein (MOG), or with a mixture of these proteins. This process triggers an exacerbated autoimmune response that culminates in demyelination of axonal tracts [82,83,84]. The degree of impairment in the animals is assessed using a neurological dysfunction scale that measures motor deficits, weakness and/or paralysis in the tail and limbs, as well as posture and gait. Approximately two weeks after the inoculation of the antigen or autoreactive T cells, peak disease severity is reached.

This model focuses on the autoimmune response mediated by T lymphocytes, which leads to acute and short-term inflammation. However, it fails to reflect key neurological aspects of the disease, such as the blockade in the maturation of the oligodendrocyte lineage, chronic axonal degeneration, and glial dysfunction. Human multiple sclerosis involves not only demyelination, but also neurodegeneration and defective remyelination—processes that are not fully replicated in the EAE model. Additionally, the disease progression in this model is uniform and does not capture the variability or unpredictable course of multiple sclerosis in humans, which may present as a relapsing–remitting or progressive pattern.

On the other hand, the severity and symptoms observed may vary depending on the animal strain used, which limits the extrapolation of the results to the human population and reduces the overall applicability of the findings.

#### 2.1.2. Theiler’s Murine Encephalomyelitis Virus Model

This model is based on the infection of mice (SJL/J, C57BL/6) with Theiler’s Murine Encephalomyelitis Virus, an RNA virus from the Picornaviridae family specific to rodents [85,86]. The infection induces chronic encephalomyelitis, triggering a sustained immune response that leads to chronic inflammation and demyelination. The exact mechanism by which a viral infection can trigger an autoimmune pathology is still not fully understood.

Following infection, an acute phase is observed, characterized by episodes of demyelination and remyelination, with a particular tropism towards the spinal cord.

Theiler’s Murine Encephalomyelitis Virus model is used in multiple sclerosis research due to its ability to replicate the autoimmune response. However, it has significant limitations. First, while it allows the study of the disease’s inflammatory mechanisms, it does not fully capture its complexity, as it fails to reflect key neurological aspects. Additionally, being a viral model, the induced demyelination affects how myelin regeneration can be assessed. In fact, remyelination in Theiler’s virus model is less efficient and more limited than in multiple sclerosis [87,88], which reduces its usefulness for testing myelin repair therapies.

In Theiler’s viral model, following the acute phase, a chronic phase develops involving demyelination–remyelination processes; however, remyelination is spontaneous, limited, and in many cases virtually absent, which limits its usefulness in studies on therapies for myelin repair [88].

Lastly, this model only represents the early stages of the disease and does not allow for the study of its long-term progression [89], limiting its applicability in researching treatments for advanced forms of multiple sclerosis.

### 2.2. Toxicity-Induced Models

These models enable controlled demyelination through the use of chemical substances commonly administered to mice of the C57BL/6J strain, which allows the study of spontaneous OPC differentiation and myelin regeneration. However, remyelination is not always complete or functionally effective [90,91,92,93,94], particularly when exposure to the drug is prolonged. In addition, myelin damage results from the toxicity of a drug that can also affect other organs, which does not accurately reflect multiple sclerosis physiopathology. Therefore, although useful, these models do not precisely replicate the pathological mechanisms of myelin-related diseases.

#### 2.2.1. Cuprizone Model

Cuprizone is a substance that disrupts copper metabolism in the body, leading to damage in the nervous system and other organs. Although copper is an essential metal for various biological functions, at high concentrations it becomes toxic to oligodendrocytes.

The exact mechanism of action of cuprizone has not been fully elucidated and it is believed that copper chelation induces severe effects related to oxidative stress and cellular damage [95,96]. When administered to mice, these effects can manifest as disorders including growth retardation, paralysis, and pregnancy termination [97,98]. Additionally, cuprizone causes inflammation and damage in the CNS associated with demyelination.

Cuprizone is commonly administered in C57BL/6 mice for 4 to 6 weeks to study acute demyelination, or up to 12 weeks for chronic demyelination. Occasionally, follow-up is performed after its withdrawal to observe remyelination. Despite continuous exposure, endogenous repair processes such as remyelination are activated, although this is limited [99,100]. The main characteristic of the model is oligodendrocyte death and demyelination in brain regions such as the corpus callosum, cerebellar peduncles, and hippocampus [101,102,103]. This process is accompanied by astrogliosis and microgliosis, which begin simultaneously with demyelination [99,104,105].

Although cuprizone withdrawal allows some remyelination, behavioral and structural alterations, axonal loss, and degeneration of the corpus callosum tracts persist, affecting learning and motor function [90,91,92,93,100,106].

After withdrawal, remyelination occurs spontaneously, but its speed and effectiveness depend on the age of the animals: in young mice, it is almost complete, while in older animals it is slower and incomplete, reflecting a limited repair capacity [107]. Additionally, prolonged exposures to cuprizone can induce chronic demyelination processes with insufficient repair, preventing full recovery of neurological function [107,108].

While the cuprizone model allows the study of demyelination and certain repair mechanisms, it does not fully replicate the neurological component of multiple sclerosis, as it fails to reflect the blockage in oligodendrocyte maturation, one of the primary barriers to remyelination in patients.

#### 2.2.2. Lysolecithin Model

Lysolecithin is a toxic substance that acts as a biological solvent, incorporating itself into the plasma membranes of oligodendrocytes and other cell types, increasing their permeability and disrupting the structure of myelin lipids [49,109]. As a result, myelin is completely degraded, which is reflected by the disappearance of its characteristic markers [81,110]. In addition to causing direct damage, lysolecithin generates an inflammatory response in the affected area. As seen in other toxic-induced demyelination models, the damage phase is followed by a repair process aimed at restoring myelin through remyelination.

This model does not fully replicate the complexity of multiple sclerosis, as demyelination occurs due to direct chemical damage rather than the biological mechanisms underlying the disease [110].

## 3. Neurological Models

Current research aims to better understand myelin regeneration and develop more accurate animal models for studying multiple sclerosis. Since oligodendrocyte differentiation is critical for repairing damage in multiple sclerosis, experimental models are needed that enable the analysis of this process and the design of more effective therapies that address the neurological aspects of the disease.

### Ras-Related (R-Ras)

Proteins of the Ras superfamily are essential regulators of intracellular signaling pathways, such as PI3K/Akt and MAPK/ERK, modulating functions like proliferation, differentiation, and cell survival [111,112,113]. This superfamily is divided into several subfamilies, including the classical Ras proteins (Hras, Kras, Nras), which have been extensively studied due to their involvement in proliferation, cell cycle regulation, and survival. When they carry mutations that constitutively activate them, they can contribute to the development of various cancers (e.g., lung, colon, and pancreas) [114].

The R-Ras subfamily includes proteins such as R-RAS1 (RRas), R-RAS2 (TC21), and R-RAS3 (MRas) [115,116,117]. Although they share 55–60% amino acid sequence identity with the classical Ras proteins [116,118], they have specific functions, including regulation of cell adhesion, extracellular matrix formation, and neuronal development. While R-Ras3 is the most highly expressed in the brain, recent studies have shown that R-Ras1 and R-Ras2 are essential for oligodendrocyte maturation and myelination processes in the central nervous system (CNS), particularly in the optic nerve. The blockade in oligodendrocyte precursor cell (OPC) maturation suggests that the molecular signals regulating myelination are disrupted, preventing proper myelin repair. In this context, R-Ras-deficient mouse models emerge as powerful tools for understanding disease progression and fostering the development of therapies aimed at promoting myelin regeneration and restoring oligodendrocyte function.

In our laboratory, three mouse models have been developed that accurately reproduce the neurological symptoms observed in multiple sclerosis and other demyelinating pathologies. These models, based on the absence of the R-Ras1 and/or R-Ras2 GTPases, have proven to be effective tools for studying alterations in myelination and the signaling pathways involved. They were generated in mice with a C57BL6/J genetic background and analyzed from 30 days postnatal, when myelination is already complete, allowing precise identification of any abnormalities.

The *R-Ras1^−/−^* and *R-Ras2^−/−^* models show a reduction in the oligodendrocyte population and alterations in CNS myelination due to decreased activation of the PI3K/Akt/mTOR and ERK1/2/MAPK pathways, which are essential for oligodendrocyte maturation and myelin formation. The absence of these GTPases leads to an increase in immature oligodendrocytes and a decrease in myelin quantity, a deficit particularly pronounced in double-mutant *R-Ras1^−/−^*; *R-Ras2^−/−^* mice. Since myelin directly affects nerve impulse conduction velocity, these animals display slower conduction, hypomyelination, and mitochondrial and axonal alterations that ultimately result in axonal degeneration.

Mitochondrial changes include variations in the number and size of mitochondria and an increase in the proteins of complexes UQCRC2, COXIV, and ATP synthase, associated with elevated ATP production [48]. This contributes to oxidative stress and protein oxidation—processes linked to the degeneration characteristic of demyelinating diseases such as MS. These phenomena are reflected in thinner, hypomyelinated axons showing signs of axonal damage, along with astrogliosis, microgliosis, and axonal dystrophy, as well as increased apoptosis of retinal ganglion cells [48,119,120].

Likewise, the absence of R-Ras1 and R-Ras2 disrupts the phosphorylation/dephosphorylation of cytoskeletal components (e.g., SMI-32, Tau1) and increases levels of PSA-NCAM, a protein associated with demyelinating pathologies. These alterations indicate axonal damage and compromise neuronal viability, as evidenced by increased apoptosis in retinal ganglion cells in double mutant mice [48]. To assess the functional impact, visual and motor tests were performed: the double knockouts showed slower responses in the optokinetic reflex and gait abnormalities, such as erratic steps and reduced stride length, compared with controls and single mutants, reflecting the impact of hypomyelination on motor function [48].

Ras-related mouse models enable the study of myelin diseases for several key reasons. First, these models lack a functional immune system. TC21, a small GTPase encoded by R-Ras2, constitutively interacts with both T cell antigen receptors and B cell antigen receptors. Consequently, *R-Ras2^−/−^* mice exhibit lymphopenia, likely due to reduced homeostatic proliferation and impaired survival of T and B cells [121]. This characteristic allows for the elimination of variability associated with the immune response observed in other models, such as immunological ones. Second, the myelin deficit in these models occurs without the need for exposure to toxins, making them more representative of myelin diseases. Third, mutant mice exhibit a blockage in oligodendrocyte maturation, along with signs of axonal damage such as astrogliosis, microgliosis, mitochondrial dysfunction, axonal degeneration, and loss of function—features also observed in human disease [48,119,120]. Finally, these models display varying degrees of hypomyelination, allowing the study of different disease stages and allowing the analysis of therapies targeting specific phases of the condition. Three genetic knock-out (KO) mice models are proposed, which faithfully replicate the neurological symptoms of patients with myelin diseases.

These models are mutant mice lacking R-Ras1 and/or R-Ras2 [119]. Specifically, the R-Ras1KO model exhibits mild hypomyelination, the R-Ras2KO model shows a moderate degree, and the double KO model (R-Ras1KO; R-Ras2KO) reflects a severe myelin impairment, suggesting an additive effect in the regulation of myelination and aligning with the most severe disease manifestations. The shades in disease severity across these models facilitates the evaluation of potential treatments at various stages of myelin damage (Table 1).

In this way, Ras-related models represent a crucial advancement in multiple sclerosis research by providing an experimental framework that accurately reflects key aspects of the pathology, such as hypomyelination, axonal damage, and the blockage in oligodendrocyte maturation. Their ability to replicate varying degrees of severity and evaluate personalized therapies positions them as a strategic tool for addressing the clinical heterogeneity of the disease and designing more effective interventions (Table 2).

## 4. New Perspectives in Drug Development

The development of drugs for the CNS is a rapidly growing area within the pharmaceutical industry, with a 30% expansion in recent years, making it the second most significant therapeutic area after oncology. In neurology, the drug development process faces considerable challenges, as the success rate in the preclinical phase is well below that of other therapeutic areas [122]. One of the primary factors contributing to the failure of these treatments is a lack of efficacy, highlighting the need for continued progress in understanding diseases of the nervous system.

In the specific case of multiple sclerosis, basic research has revealed that it has a complex etiology, influenced by a significant neurological component and associated with genetic factors, such as slight increases in the expression of certain genes, as well as environmental factors, including Epstein–Barr virus (EBV) infection, low exposure to ultraviolet B (UVB) light, obesity, vitamin D deficiency and smoking (Figure 2) [50,123,124,125,126]. Currently, research efforts are focused on unraveling the molecular foundations responsible for the myelination process, while also developing and validating animal models that accurately replicate the disease’s distinctive features. This aims to advance the understanding of its pathophysiology and support the search for effective therapies.

Some studies indicate that understanding how oligodendrocytes are regenerated and replaced is crucial for myelin regeneration [127,128,129,130]. Although the importance of neurological factors in multiple sclerosis is undeniable [131,132,133,134], current treatments focus on modulating the immune response without directly addressing myelin regeneration or the restoration of neuronal functionality. To address this, the pharmaceutical industry has shifted toward developing therapies that promote oligodendrocyte differentiation and the regeneration of the myelin sheath [135,136,137]. To ensure the effectiveness of new compounds, it is essential to have animal models that accurately replicate the neurological pathophysiology of multiple sclerosis and enable precise evaluation of the efficacy of these new therapeutic compounds before their transition to the clinical phase [138].

Classical models, such as experimental autoimmune encephalomyelitis or those induced by viruses or toxins, have significant limitations, as they emphasize inflammation but fail to replicate the neurological component of myelin diseases. In particular, these models do not effectively allow for the evaluation of oligodendrocyte maturation or the precise assessment of a drug’s ability to restore myelin in a relevant pathophysiological environment. This restricts their usefulness for testing new therapies aimed at myelin regeneration and complicates the extrapolation of results into the clinical phase [138].

As a result of these limitations, a notable discrepancy has been observed between the results obtained in experimental models and those in clinical trials. Numerous therapeutic strategies that showed promising effects in the preclinical phase have consistently failed to demonstrate clinical benefits (http://www.patentinspiration.com, https://www.lens.org/ (accessed on 2 April 2025)), highlighting a significant gap in translating laboratory findings into medical practice.

In this context, animal models related to Ras represent a powerful tool for the pharmaceutical industry, as they allow for direct analysis of a drug’s ability to induce myelination. In these models, the reduction and blockade in oligodendrocyte maturation facilitate the evaluation of compounds that promote cell differentiation and myelin regeneration.

Within the typical sequence of preclinical development for new treatments, R-Ras models occupy an intermediate stage in this process, prior to potential transition into clinical phases. R-Ras-based animal models could represent progress in this regard, as by more accurately replicating the neurological features of myelin diseases, they offer the opportunity to evaluate not only toxicity but also the pharmacological efficacy of potential treatments, thus moving us closer to a more predictive and clinically relevant model.

**Figure 3:**
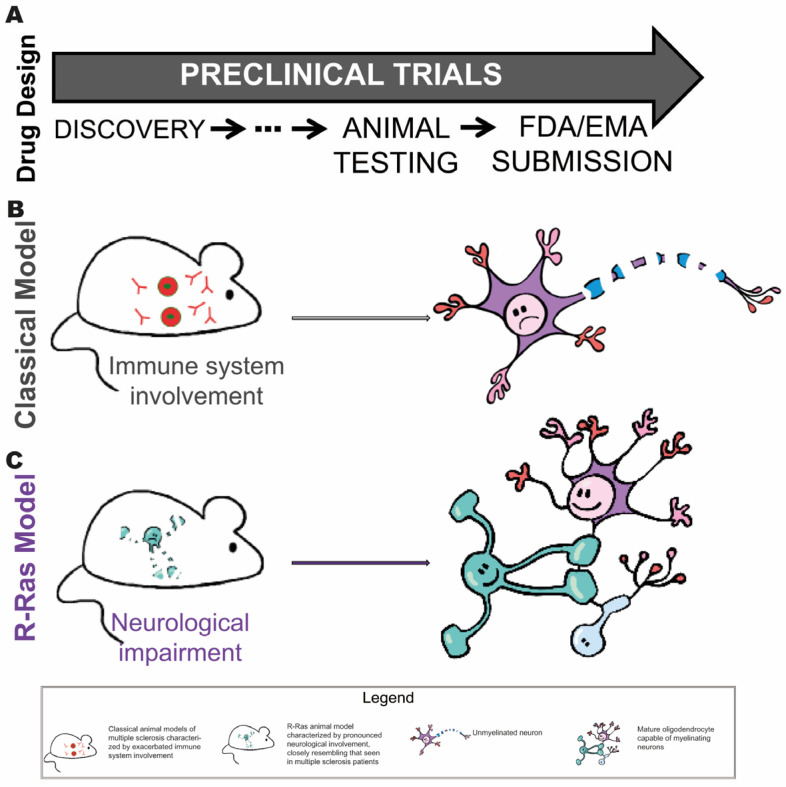
**Stages of drug development where the Ras-related model could be useful.** (**A**) Summary of the preclinical process: from target identification and in vitro evaluation, through testing in animal models, to submitting the dossier to regulatory agencies (FDA/EMA) for authorization to initiate human clinical trials. The schematic of the preclinical process provides a simplified view of the typical stages in the preclinical development of new treatments, highlighting the key role that animal models play in the preclinical validation phase. (**B**) In traditional animal models used in preclinical drug development, the emphasis is generally placed on toxicity assessment. However, these models rarely allow accurate prediction of therapeutic efficacy under human conditions. (**C**) R-Ras-based animal models could represent progress in this field by enabling the evaluation not only of toxicity, but also of the pharmacological efficacy of potential treatments.

The use of these models in preclinical research can significantly improve the selection of therapeutic candidates, optimizing resources and increasing the likelihood of success in developing effective treatments for multiple sclerosis and other myelin-related diseases (Figure 3).

## 5. Conclusions

Myelin diseases, such as multiple sclerosis, cause oligodendrocyte death, myelin loss and neurodegeneration leading to sensory, motor and cognitive deficits. Current treatments primarily focus on controlling immune response. A growing focus on regenerative strategies now aims to stimulate oligodendrocyte differentiation. This study highlights Ras-related animal models for evaluating compounds that promote myelination. We present the usefulness of Ras-related animal models for evaluating the efficacy of compounds that promote oligodendrocyte differentiation. In these models, the absence of functional oligodendrocytes allows for a direct assessment of the drugs’ ability to induce myelination.

## Figures and Tables

**Table 1:** **Differential characteristics between classical models and Ras-related models**. This table summarizes various animal models used to study myelin-related diseases such as multiple sclerosis, highlighting key phenotypic features associated with these pathologies. Green cells indicate phenotypic aspects that are present or well-reproduced in the corresponding model, while red cells indicate aspects that are not covered or are absent.

	Immunological Models		Toxic Models		Neurological Models		
	EAE	TMEV	Cuprizone	Lysolecithin	*R-Ras1^−/−^*	*R-Ras2^−/−^*	*R-Ras1^−/−^; R-Ras2^−/−^*
Myelin deficit	YES	YES	YES	YES	YES	YES	YES
Oligodendrocyte maturation blockage	NO	NO	NO	NO	YES	YES	YES
Myelin sheath blockage	NO	NO	NO	NO	YES	YES	YES
Energetic and metabolic disturbances	NO	NO	NO	NO	YES	YES	YES
Neuronal death	YES	YES	YES	YES	YES	YES	YES
Sensory, motor and cognitive disturbances	YES	YES	YES	YES	YES	YES	YES
	**Classical models**				**New Approaches**		

**Table 2:** **Differential phenotypic characteristics of R-Ras mouse models.** The table shows the three R-Ras mouse models that replicate the neurological symptoms characteristic of multiple sclerosis patients. The different R-Ras mutant mice display varying degrees of symptom severity, which facilitates the study of the different stages of the disease. Specifically, the *R-Ras1^−/−^* model exhibits a mild degree of hypomyelination, the *R-Ras2^−/−^* model presents a moderate degree, and the deletion of both proteins in the double knockout model *R-Ras1^−/−^; R-Ras2^−/−^* results in a severe degree of hypomyelination. The first column indicates the altered events. The arrows (↑/↓) indicate an increase or decrease compared with control (wild-type) mice. The equals sign (=) indicates no relevant changes compared with the control. ↑, ↑↑, and ↑↑↑ represent mild, moderate, or severe increases, respectively; likewise, ↓, ↓↓, and ↓↓↓ represent mild, moderate, or severe decreases, respectively.

Disease Features	*R-Ras1^−/−^*	*R-Ras2^−/−^*	*R-Ras1^−/−^; R-Ras2^−/−^*
Oligodendrocyte population	↓	↓↓	↓↓↓
Immature oligodendrocytes	↑	↑↑	↑↑↑
Mature oligodendrocytes	↓	↓↓	↓↓↓
Myelinated axons	↓	↓↓	↓↓↓
Channel disposition	↑	↑↑	↑↑↑
Conduction velocity	↑	↑↑	↑↑↑
Mitochondrial: Number Size Channel proteins Respiration	↑ ↑ ↑ ↑	↓↓ = = =	↓↓↓ ↑↑↑ ↑↑↑ ↑↑↑
Metabolism	Altered	Altered	↑↑↑
Astro & microgliosis	=	=	↑↑↑
Axonal cytoskeleton alterations	↑	↑↑	↑↑↑
Motor function	↓	↓↓	↓↓↓
Visual function	↓	↓↓	↓↓↓
**Resultant phenotype**	**Mild**	**Moderate**	**Severe**

Legend: ↓: Mild decrease, ↓↓: moderate decrease, ↓↓↓: severe decrease, =: unaltered, ↑: mild increase, ↑↑: moderate increase, ↑↑↑: severe increase.

## Data Availability

This study did not generate any new data. All data analyzed in this study are from previously published studies cited in the references.

## Abbreviations

The following abbreviations are used in this manuscript:

OPC	Oligodendrocyte Progenitor Cell
DMT	Disease-Modifying Therapy
EAE	Experimental Autoimmune Encephalomyelitis
MBP	Myelin Basic Protein
PLP	Proteolipid Protein
MOG	Myelin Oligodendrocyte Glycoprotein
KO	Knock-Out
